# Effects of Different Hot Working Techniques on Inclusions in GH4738 Superalloy Produced by VIM and VAR

**DOI:** 10.3390/ma11061024

**Published:** 2018-06-15

**Authors:** Zhengyang Chen, Shufeng Yang, Jingshe Li, Hao Guo, Hongbo Zheng

**Affiliations:** 1 School of Metallurgical and Ecological Engineering, University of Science and Technology Beijing, Beijing 100083, China; chenzhengyang@xs.ustb.edu.cn (Z.C.); lijingshe@ustb.edu.cn (J.L.); guohaoustb@sina.com (H.G.); hongbozhengp@hotmail.com (H.Z.); 2 Beijing Key Laboratory of Special Melting and Preparation of High-End Metal Materials, Beijing 100083, China

**Keywords:** superalloy, melting, hot working, inclusion

## Abstract

Hot working is a key process in the production of superalloys; however, it may result in the formation of inclusions that affect the superalloy performance. Therefore, the effects of hot working on inclusions in a superalloy must be studied. GH4738 superalloy was manufactured, herein, by vacuum induction melting and vacuum arc remelting. Hot working was performed by unidirectional drawing, upsetting and drawing, and upsetting/drawing with radial forging. The types and distributions of inclusions after these three hot working processes and those in an original ingot were analyzed using scanning electron microscopy, energy dispersive spectroscopy, and Image-Pro Plus software. The results showed that the melting technology essentially determined the inclusion types in GH4738. Four types of inclusions were found in the experiments: TiC–TiN–Mo–S composite, TiC–TiN composite, Ce–Mo–S composite, and SiC inclusions. In the case of hot working by unidirectional drawing, the average inclusion size first decreased, and then increased from the center to the edge. In the case of upsetting and drawing, and upsetting/drawing with radial forging, the average inclusion size decreased from the center to the edge.

## 1. Introduction

The temperature and mechanical performance requirements of key engine components, such as turbine disks and blades [1,2,3,4,5], are becoming more stringent as aerospace engines are moving toward better reliability, higher thrust-to-weight ratio, and larger size. Superalloys that afford excellent high-temperature strength, good oxidation resistance, high hot corrosion resistance temperature, etc. [6,7,8], have become indispensable materials for manufacturing aerospace engine components, and they are considered to provide “a new generation of superalloys for every generation of aircraft” [9,10,11].

However, the performance and structural integrity of superalloys can be greatly affected by inclusions. Thus far, researchers have controlled inclusion growth in two ways. Some researchers have modified melting technologies to improve the purity of superalloys, and thereby enhance their performance. For example, Degawa et al. [12] prepared IN738 and MarM247 alloys by vacuum induction melting (VIM) in a CaO crucible; they found that ingot quality was improved significantly, and that ingots contained less than 10 ppm of N, O, and S (each). Schneider et al. [13] found that using a low-frequency alternating current instead of direct current for electroslag remelting resulted in higher ingot purity. Shevchenko et al. [14] demonstrated that the time variation and asymmetric distribution of the arc in vacuum arc remelting (VAR) did not facilitate slag discharge by the molten pool, thus affecting ingot purity. Appropriate arc control could effectively improve the ingot quality.

Other researchers improved alloy properties by studying and manipulating the type, size, morphology, distribution, and evolution of inclusions [15,16,17]. Wang et al. [18] analyzed inclusions at different positions in a 20Cr13 hot-rolled stainless-steel round bar, and identified changes in inclusions from the edge to the center, thereby enabling growth control and elimination of inclusions in hot-rolled bars. Kawakami et al. [19] studied the generation mechanisms of nonmetallic inclusions in high-cleanliness steel, and found that reducing the inclusion size during secondary refining effectively enhanced alloy properties. Jiang et al. [20] studied the evolution mechanisms of nonmetallic inclusions in high-strength steel alloys, and found that the steel-slag reaction time strongly influenced the type, composition, and shape of inclusions. These studies suggested that appropriate control over the inclusion type and morphology could improve the fatigue resistance of alloys.

Thus far, however, few studies have reported on the types and distributions of inclusions formed in superalloys, and on the effects of hot working techniques on inclusions in superalloys. Inclusions with different sizes and distributions formed in the superalloy after hot working could lead to different fatigue strengths [21]. Therefore, this study performed VIM and VAR of the GH4738 superalloy followed by hot working via unidirectional drawing, upsetting and drawing, and upsetting/drawing with radial forging. The type, percentage, size change, and distribution of inclusions in GH4738 were observed and recorded to analyze the effects of three hot working methods, and the original method, on the inclusion type and distribution. The results of this study should provide engineering guidelines, and a theoretical foundation for optimizing the melting technology of superalloys and selecting a reasonable hot working method.

## 2. Experimental

### 2.1. Melting and Hot Working

Electrolytic nickel plate, high-purity chromium, high-purity molybdenum, some GH4738 revert materials, and other elemental alloys were used as raw materials in this experiment. The GH4738 superalloy was melted by VIM (Consarc, Rancocas, NJ, USA), and the vacuum degree of the whole melting process was kept at 2 Pa. Argon was injected before adding trace elements, such as Mg, B, and Zr, to prevent volatilization, and its effect on alloy composition accuracy. Finally, four ingots of Φ460 mm were poured at 1400 °C, and then, they were cooled for 3 h in the vacuum induction furnace.

After removing risers, the four ingots were polished to prevent secondary pollution caused by refractory and surface oxide coatings before VAR. The four ingots were then melted by VAR (ALD, Hanau, Germany). The initial melting rate was set at 8 kg/min to form a molten pool quickly. Then, the melting rate in the stable melting stage was controlled at a constant melting speed, and gradually reduced to 4.5 kg/min. Toward the end of the process, the voltage and current were adjusted to gradually reduce the melt rate and provide a controlled hot top. Vacuum degree of less than 1 Pa was used during the entire melting process. Each ingot was cooled for 2 h in the vacuum arc furnace after VAR. Finally, we obtained four superalloy bars of Φ508 mm. Table 1 shows their chemical composition.

**Figure 1:**
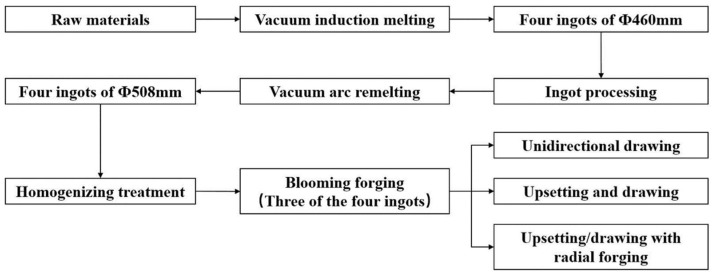
Roadmaps of melting and hot working.

The four superalloy bars were homogenized in a heat treatment furnace before hot working. In the heat treatment furnace, the four superalloy bars were heated to 1000 °C for 5 h, left to stand for 10 h, and finally cooled to 400 °C. Three of the four ingots were then subjected to different hot working techniques: unidirectional drawing (Φ508 mm → Φ470 mm → Φ430 mm → Φ400 mm → Φ350 mm → Φ290 mm → Φ220 mm), upsetting and drawing (Φ508 mm → Φ470 mm → Φ430 mm → Φ400 mm → Φ430 mm → Φ400 mm → Φ350 mm → Φ290 mm → Φ220 mm), and upsetting/drawing with radial forging (Φ508 mm → Φ470 mm → Φ430 mm → Φ400 mm → Φ430 mm → Φ400 mm → Φ350 mm → Φ300 mm → Φ240 mm → Φ230 mm → Φ220 mm) by a high-speed forging hydraulic machine and a radial forging machine, to yield bars of a uniform size (Φ220 mm). The superalloy bars were subjected to heat treatment (850 °C for 4 h) after each plastic deformation. Figure 1 shows the roadmaps of melting and hot working.

### 2.2. Preparation and Testing

**Figure 2:**
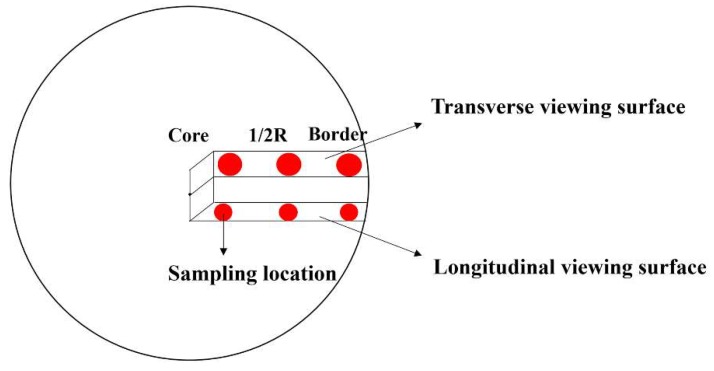
Schematic diagram of sample position.

The 15 mm × 15 mm × 15 mm samples used in the experiments were cut from the center from a point at a distance of 1/2*R* (*R* is the radius) from the center, and from the edges of the transverse and longitudinal sections of the bars. Figure 2 shows the sampling positions.

The metal samples were mechanically polished. The inclusion size, morphology, and composition were characterized using a scanning electron microscope–energy dispersive spectrometer (SEM–EDS, SEM, Phenom proX scanning electron microscopy, Eindhoven, The Netherlands). Each sample was divided into four zones to obtain more accurate inclusion data. Twenty-five different viewing fields were selected in each zone to perform statistical analyses. Image-Pro Plus software (Version 6.0, Media Cybernetics, Inc., Rockville, MD, USA) was used to analyze the average size and distribution of inclusions.

## 3. Results and Discussions

### 3.1. Inclusion Type and Percentage Contribution

**Figure 3:**
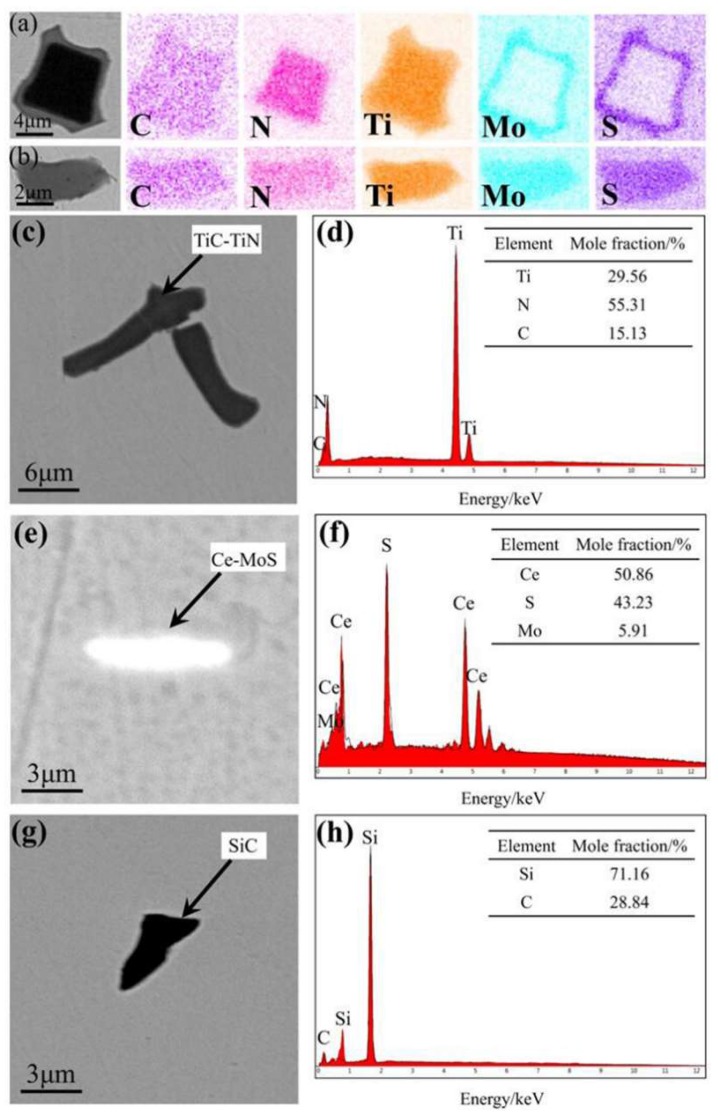
SEM images and EDS mapping of dominant inclusions. (**a**,**b**) TiC–TiN–Mo–S; (**c**,**d**) TiC–TiN; (**e**,**f**) Ce–Mo–S; (**g**,**h**) SiC.

**Figure 4:**
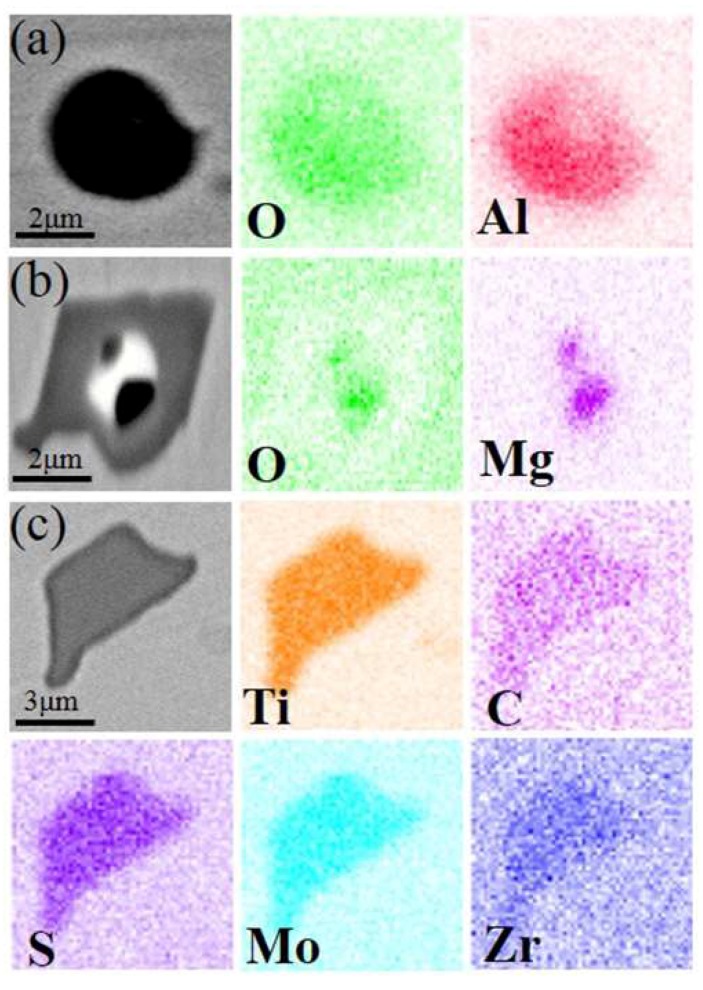
SEM images and EDS mapping of other inclusions: (**a**) Al_2_O_3_; (**b**) MgO; and (**c**) Ti–Mo–Zr–C–S.

The data collection and analysis of the types and percentages of inclusions in the original sample and those obtained using the three different hot working methods showed nearly identical results, indicating the minute influence of the different hot working techniques on the inclusion type and percentage (Table 2). Therefore, the melting technology should be considered in the data analysis. The inclusion types and percentages in the samples were obtained using only one hot working method were analyzed in detail, because of the length restrictions of this paper (Figure 3 and Figure 4).

Five different types of inclusions can be identified in the samples shown in the table and two figures, of which four are dominant: TiC–TiN–Mo–S composite, TiC–TiN composite, Ce–Mo–S composite, and SiC inclusions. The TiC–TiN–Mo–S composite inclusions had the highest proportion, and existed in two different forms (Figure 3a,b). The composite inclusions could have been formed during superalloy melting because different types of inclusions collided, combined, or were enriched at the surface because of the circulation and convection of the molten metals and the different densities of various inclusions [22].

Figure 3c,d show that composite inclusions were formed by both TiC and TiN. TiN had a lower standard Gibbs free energy of formation than TiC; therefore, it preferentially nucleated. TiC then precipitated on the TiN surface, aggregated, and grew, forming TiC–TiN composite inclusions. Both compounds had face-centered cubic structures with similar lattice constants, and they easily formed solid solutions during their growth. Therefore, no clear nucleation center was observed [23].

An analysis of Figure 3e,f revealed no Ce among the constituents of GH4738; however, Ce–Mo–S inclusions were found in the ingots. These inclusions were caused by the inadequate purity level of some revert or raw materials fed during VIM of GH4738, which led to Ce contamination and formation of Ce–Mo–S inclusions.

Figure 3g,h show that during VIM of GH4738, C was added into the furnace as a deoxidizing agent to form CO, thereby removing oxygen. Less CO was produced as the oxygen concentration gradually decreased in the liquid metal. A part of this could not be released because of the small bubble size, and it remained attached to the crucible wall, thereby facilitating a reaction between a fraction of CO with Si in the crucible wall material and producing SiC inclusions [24].

The remaining inclusion types had smaller proportions. Although the number of undesirable elements could be kept low during VIM and VAR of GH4738, some trace elements were still segregated to form inclusions (Figure 4a–c).

The earlier analyses showed that the three hot working methods had minute influences on the inclusion types and percentages in GH4738. The raw material purity, VIM refining time, slag discharge extent of metal molten pool in VAR, etc., were the determining factors.

### 3.2. Distribution of Inclusions in Transverse and Longitudinal Directions

**Figure 5:**
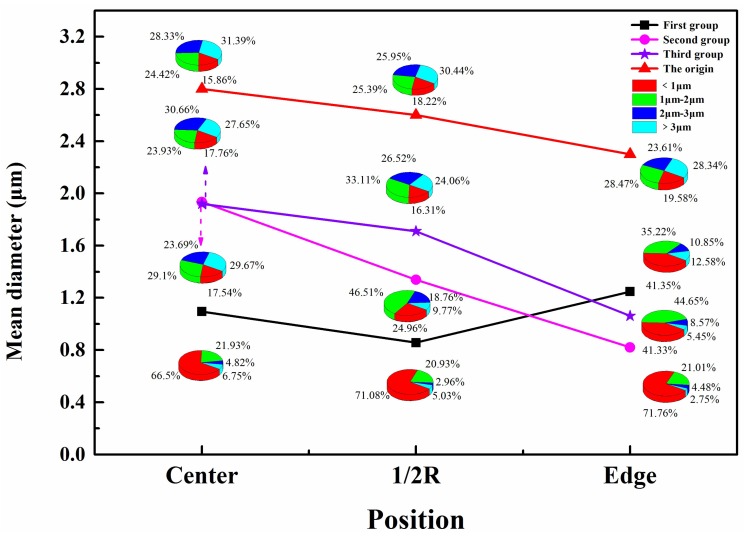
Inclusion distributions in transverse direction for samples obtained using three hot working methods and original sample.

**Figure 6:**
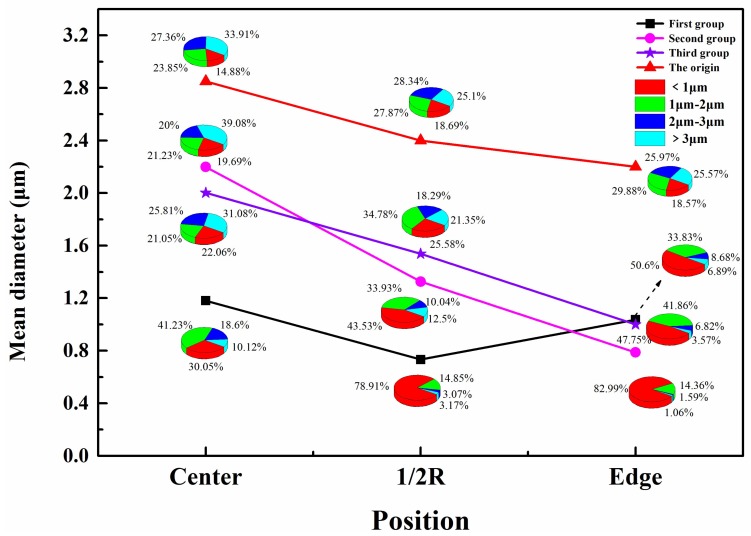
Inclusion distributions in longitudinal direction for samples obtained using three hot working and original sample.

Figure 5 and Figure 6 show the distributions of the inclusions along the transverse and longitudinal directions, for the original sample and the samples obtained using the three hot working methods. Analyzing both figures led to the following result: in the original ingot, a moderate increase in the percentage of inclusions with sizes less than 2 μm from the center to the edge resulted in the average inclusion size gradually decreasing. This decrease was caused by the gradual decrease in the cooling rate of the ingot from the edge to the center during VAR, resulting in less time for inclusion polymerization or inclusion growth owing to particle collisions. Therefore, the average inclusion size at the edge was the smallest, whereas that at the center was the largest. After the ingots were subjected to the three different hot working methods from the center to 1/2R and to the edge, they showed a decrease in the average inclusion size in that direction. The inclusions deformed and fractured or broke as the deformation of the ingot increased in the hot working process, thereby resulting in the average inclusion size decreasing [25]. This study analyzed the effects of three different hot working methods on superalloy inclusions, because such methods affect the inclusion distribution.

The average inclusion size for hot working by unidirectional drawing (first group) first decreased, and then increased from the center to the edge. At 1/2*R*, inclusions with sizes less than 2 μm had the highest percentage, resulting in the lowest average inclusion size at that spot. According to the classical plasticity theory [26], deformation rarely occurs in the contact region between the forged piece and the plate; thus, inclusions in this region do not undergo much deformational fracture when force is applied. The average inclusion size at the edge was larger compared to that at 1/2*R*. As noted in a previous study [27], for hot working by unidirectional drawing, the average chord length of grains at the center was greater than that at 1/2*R*, indicating that a larger deformation force was experienced by the inclusions at 1/2*R* compared to that at the center (i.e., large inclusions underwent deformational fracture or were more readily broken into numerous small inclusions). Therefore, the average inclusion size was smaller at 1/2*R* than at the center. In conclusion, hot working by unidirectional drawing primarily affects the inclusion size at the 1/2*R* position in the superalloy.

In hot working with upsetting and drawing (second group), the sharp increase in the percentage of inclusions with sizes less than 2 μm from the center to 1/2*R* and to the edge resulted in a rapid decrease in the average inclusion size in that direction. This decrease was caused by the large deformation energy generated in the forged piece during repeated upsetting and drawing, making it difficult for the temperature of the whole work piece to decrease. The alloy structure at the center had a lower forging penetration efficiency and degree of deformation as it cooled more slowly [28]. The internal inclusions there underwent less deformational fracture or breaking and, as such, had the highest average size. However, the multidirectional deformation caused by repeated upsetting and drawing also intensified the degree of fracture and breaking among the inclusions at 1/2*R* and along the edge, making some of them more dispersed in distribution [29]. Furthermore, cooling occurred much faster at the edge than at 1/2*R*, thereby facilitating the dispersion of fine inclusions. Therefore, the average inclusion size was the smallest at the edge. We can conclude that hot working by upsetting and drawing mainly affected the inclusion size at the superalloy edge.

The percentages of inclusions of different sizes at the center, at 1/2*R*, and along the edge showed a significant increase for inclusions of size less than 2 μm when the ingots were subjected to upsetting/drawing with radial forging during hot working (third group), forming a trend of decreasing average inclusion size from the center to the edge. As noted in a previous study [30], the grain size was the finest at the edge for a work piece subjected to radial forging. In other words, the effect of radial forging was mainly seen in the grain size at the edge, and the grain refinement at the edge was accompanied by a reduction in the inclusion size there. Considering the variations in the average inclusion size from the center to the 1/2*R* point and to the edge of a forged piece subjected to upsetting and drawing, as described in the previous section, the third hot working method appears to have chiefly affected the inclusion size at the superalloy edge.

## 4. Conclusions

The size and distribution of inclusions formed after superalloy melting had thus far been unclear. A large difference was found between the inclusions and the matrix, causing different fatigue strengths in superalloys owing to the different sizes and distributions of inclusions in each region after applying different hot working methods, which affected the superalloy performance. The influence of inclusions in the superalloy and on their properties must be reduced, and the performance degradation induced by inclusions formed in different hot working methods must be avoided. Therefore, this study analyzed the effects of three different hot working techniques, namely, unidirectional drawing, upsetting and drawing, and upsetting/drawing with radial forging, on inclusions in GH4738 superalloy. GH4738 was fabricated by VIM and VAR. Hot working was also performed. The type, percentage, size change, and distribution of inclusions in GH4738 were observed and recorded, to analyze the effects of the three hot working methods on the inclusion type and distribution. This study provides engineering guidelines and a theoretical foundation for optimizing the melting technology of superalloy, and selecting a reasonable hot working method. The following conclusions were drawn from this study:

1. The three hot working methods have minimal impact on the inclusion type and percentage in GH4738. The melting technology plays a determining role. Four types of inclusions were found in the superalloy during this experiment: TiC–TiN–Mo–S composite, TiC–TiN composite, Ce–Mo–S composite, and SiC inclusions.
2. For GH4738 subjected to hot working by unidirectional drawing, the average inclusion size first decreased, and then increased from the center to the edge. This technique primarily affected the inclusion size at the 1/2*R* point of the superalloy.
3. For GH4738 subjected to hot working by upsetting and drawing, and upsetting/drawing with radial forging, the average inclusion size decreased from the center to the edge. These two techniques primarily affected the inclusion size at the edge of the superalloy.

## Figures and Tables

**Table 1:** Chemical composition of the GH4738 superalloy (%).

Cr	C	Co	Mo	Al	Ti	S	Ni
18.92	0.07	13.13	3.96	1.47	2.97	<0.15	Balance

**Table 2:** Proportions of various types of inclusions for three hot working and origin methods (%).

Method	TiC–TiN–Mo–S	TiC–TiN	Ce–Mo–S	SiC	Others
**Unidirectional Drawing**	75.2	8.9	7	6.9	2
**Upsetting and Drawing**	75	9	7	7	2
**Upsetting/Drawing with Radial Forging**	75.4	9.2	6.8	6.7	1.9
**Origin**	74.6	9.1	7.1	6.9	2.3
